# Quantitative assessment of TIM-3 polymorphisms and cancer risk in Chinese Han population

**DOI:** 10.18632/oncotarget.8157

**Published:** 2016-03-17

**Authors:** Xueren Gao, Jiaojiao Yang, Youji He, Jianqiong Zhang

**Affiliations:** ^1^ Department of Microbiology and Immunology, Medical School of Southeast University, Nanjing, Jiangsu, China; ^2^ Jiangsu Key Laboratory of Molecule Imaging and Functional Imaging, Key Laboratory of Developmental Genes and Human Disease, Ministry of Education, Medical School of Southeast University, Nanjing, Jiangsu, China

**Keywords:** TIM-3, polymorphism, cancer

## Abstract

Previous studies have investigated the associations of *TIM-3* polymorphisms (−1516G/T, −574G/T, and +4259T/G) with cancer risk in Chinese Han population, but the results remain conflicting. Therefore, we conducted a meta-analysis to derive a more precise estimation of the associations. The pooled data showed that *TIM-3* polymorphisms (−1516G/T, −574G/T, and +4259T/G) were significantly associated with an increased risk of overall cancer in Chinese Han population. Subgroup analyses based on cancer system showed that *TIM-3* −1516G/T polymorphism was only associated with an increased risk of digestive system cancer in Chinese Han population. *TIM-3* −574G/T polymorphism was associated with an increased risk of digestive system cancer and other cancer in Chinese Han population. *TIM-3* +4259T/G polymorphism was only associated with an increased risk of other cancer in Chinese Han population. In summary, our results indicated that *TIM-3* polymorphisms (−1516G/T, −574G/T, and +4259T/G) were associated with the increased risk of cancer in Chinese Han population.

## INTRODUCTION

T-cell immunoglobulin and mucin domain-containing molecule 3 (TIM-3) is a type I cell surface glycoprotein and can inhibit the activation of innate immune cells, such as dendritic cells (DCs), macrophages, and natural killer (NK) cells. For instance, increased TIM-3 expression in tumor-infiltrating DCs could inhibit an innate response to nucleic acids [1]. The in vivo administration of anti- TIM-3 antibody could increase the number and activation of macrophages, suggesting that TIM-3 might inhibit macrophage activation and function [2]. In addition, TIM-3 acted as an inhibitor of macrophage activation, and blockade of the TIM-3 pathway could lead to decreased CD80 costimulatory molecule expression on macrophages and an enhanced inflammatory response [3]. NK cells protect the host against viral infection and cancer. Several lines of studies have shown that the activity of NK cells can be inhibited by TIM-3 [4,5]. For instance, in chronic hepatitis B infection, TIM-3 expression was upregulated on NK cells, which suppressed NK cells function. However, this process was reversed by blockade of the TIM-3 pathway, which supported a negative regulatory role of TIM-3 in the activity of NK cells [5]. In addition to innate immunity, TIM-3 has also been reported to involve adaptive immunity. In HCV-infected HBV vaccine non-responders, TIM-3 blockade improved IL-12p35 and inhibited IL-23p19 productions by CD14^+^ monocytes, leading to reduction of Th17 cells [6].

Human TIM-3 gene is located in chromosome 5q33.3 and contains a large number of single nucleotide polymorphisms (SNPs). Among them, the following three SNPs are common and widely studied: −1516G/T and −1516G/T polymorphisms in the promoter region and +4259T/G polymorphism in the encoding region (amino acid substitution: arginine to leucine). In 2010, the associations between *TIM-3* −1516G/T, −1516G/T, and +4259T/G polymorphisms and cancer risk were firstly reported in Chinese Han population [7]. Since then, more and more epidemiologic studies from Chinese Han population investigated the role of the three SNPs in the risk of cancer, including non-Hodgkin lymphomas (NHL), hepatocellular carcinoma, non-small-cell lung cancer (NSCLC), pancreatic cancer, and renal cell carcinoma [8–13]. However, the results are inconsistent. Furthermore, a single-center study may have an inadequate sample size and lack statistical power to obtain reliable conclusions. Thus, we performed a meta-analysis of all eligible studies to obtain a more precise estimation of the associations.

## RESULTS

### Study selection and characteristics

**Figure 1:**
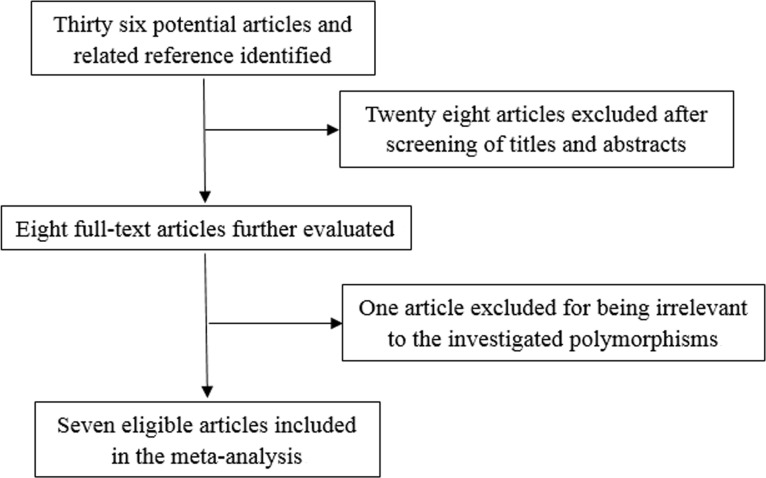
Flow chart of study selection in the meta-analysis

The study selection process is shown in Figure 1. A total of 36 articles were initially retrieved from electronic databases including PubMed, EMBASE and Chinese National Knowledge Infrastructure (CNKI). After reviewing the titles, abstracts and full text, we excluded 29 irrelevant studies. Finally, 7 articles published between 2010 and 2013 were included in the current meta-analysis. The main characteristics of all eligible studies are shown in Table 1 and S1. All the included studies were conducted in Chinese Han population. Furthermore, all of these studies assessed the association between *TIM-3* −1516G/T polymorphism and cancer risk. However, *Li Z*'s studies in 2012 and 2013 contained overlapping data. According to inclusion and exclusion criteria, *Li Z*'s study in 2013, which contained the latest and most complete data, was adopted. Finally, six articles including 2039 cases and 2372 controls were used to estimate cancer risk associated with *TIM-3* −1516G/T polymorphism. For *TIM-3* −1516G/T and +4259T/G polymorphisms, six articles with 1912 cases and 2236 controls were included.

**Table 1:** Main characteristics of all studies included in the meta-analysis

First author	Year	Country	Genotyping method	Cancer type	Source of controls	Case	Control	Plymorphisms
Song, H.	2013	China	PCR-RFLP	Non-Hodgkin lymphomas	Hospital	496	512	−1516G/T,−574G/T,+4259T/G
Li, Z.	2013	China	PCR-RFLP	Hepatocellular carcinoma	NA	271	318	−1516G/T
Bai, J.	2013	China	PCR-RFLP	Non-small-cell lung cancer	Population	432	466	−1516G/T,−574G/T,+4259T/G
Tong, D.	2012	China	PCR-RFLP	Pancreatic cancer	Hospital	306	422	−1516G/T,−574G/T,+4259T/G
Li, Z.	2012	China	PCR-RFLP	Hepatocellular carcinoma	NA	144	182	−574G/T,+4259T/G
Cai, C.	2012	China	PCR-RFLP	Renal Cell Carcinoma	Hospital	322	402	−1516G/T,−574G/T,+4259T/G
Cao, B.	2010	China	PCR-RFLP	Gastric cancer	Hospital	212	252	−1516G/T,−574G/T,+4259T/G

PCR-RFLP: PCR-restriction fragment length polymorphism; NA: not available.

### Quantitative data synthesis

**Figure 2:**
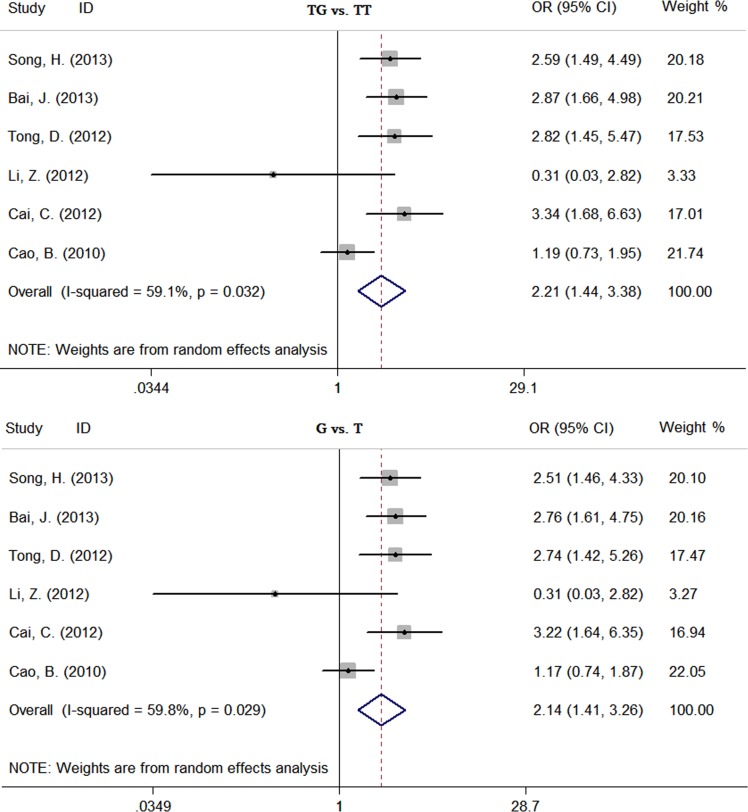
Forest plot of effect estimates for *TIM-3* +4259T/G polymorphism and overall cancer risk

The results of this meta-analysis are shown in Table 2. The pooled risk estimates indicated that *TIM-3* −1516G/T polymorphism was associated with an increased risk of overall cancer (GT *vs*. GG: OR = 1.38, 95%CI: 1.08-1.77, *P*z = 0.01; TT+GT *vs*. GG: OR = 1.40, 95%CI: 1.08-1.83, *P*z = 0.01; T *vs*. G: OR = 1.39, 95%CI: 1.07-1.79, *P*z = 0.01) (Figure S1). The similar associations were also found between *TIM-3* −1516G/T and +4259T/G polymorphisms and overall cancer risk. For *TIM-3* −1516G/T polymorphism, subjects carrying GT genotype or T allele had a significantly increased risk of overall cancer compared with those carrying the GG genotype or G allele, respectively (GT *vs*. GG: OR = 1.99, 95%CI: 1.50-2.64, *P*z < 0.01; T *vs*. G: OR = 1.95, 95%CI: 1.48-2.58, *P*z < 0.01) (Figure S2). For *TIM-3* +4259T/G polymorphism, subjects carrying TG genotype or G allele had a significantly increased risk of overall cancer compared with those carrying the TT genotype or T allele, respectively (TG *vs*. TT: OR = 2.21, 95%CI: 1.44-3.38, *P*z < 0.01; G *vs*. T: OR = 2.14, 95%CI: 1.41-3.26, *P*z < 0.01) (Figure 2). In subgroup analyses based on cancer system, we found that *TIM-3* −1516G/T polymorphism was only associated with an increased risk of digestive system cancer (GT *vs*. GG: OR = 1.75, 95%CI: 1.04-2.92, *P*z = 0.03; TT+GT *vs*. GG: OR = 1.79, 95%CI: 1.05-3.05, *P*z = 0.03; T *vs*. G: OR = 1.77, 95%CI: 1.05-2.96, *P*z = 0.03). *TIM-3* −1516G/T polymorphism was associated with an increased risk of digestive system cancer (GT *vs*. GG: OR = 1.77, 95%CI: 1.08-2.91, *P*z = 0.02; T *vs*. G: OR = 1.75, 95%CI: 1.08-2.85, *P*z = 0.02) and other cancer (GT *vs*. GG: OR = 2.11, 95%CI: 1.26-3.56, *P*z = 0.01; T *vs*. G: OR = 2.07, 95%CI: 1.25-3.44, *P*z = 0.01). *TIM-3* +4259T/G polymorphism was only associated with an increased risk of other cancer (TG *vs*. TT: OR = 2.87, 95%CI: 2.04-4.02, *P*z < 0.01; G *vs*. T: OR = 2.77, 95%CI: 1.98-3.87, *P*z < 0.01).

**Table 2:** Meta-analysis of the association between *TIM-3* polymorphisms and cancer risk in Chinese Han population

Polymorphisms	Comparison	Subgroup	Heterogeneity test		Model	*P*_Z_	*P*_E_	OR (95% CI)
			I^2^ (%)	***P***_H_				
−1516G/T	GT *vs*. GG	Overall	53.6	0.06	R	0.01	0.05	**1.38 (1.08-1.77)**
		Digestive system cancer	70.2	0.04	R	0.03		**1.75 (1.04-2.92)**
		Other cancer	0	0.89	F	0.14		1.17 (0.95-1.44)
	TT+GT *vs*. GG	Overall	58.5	0.03	R	0.01	0.06	**1.40 (1.08-1.83)**
		Digestive system cancer	72.8	0.03	R	0.03		**1.79 (1.05-3.05)**
		Other cancer	0	0.89	F	0.14		1.17 (0.95-1.44)
	T *vs*. G	Overall	60.6	0.03	R	0.01	0.06	**1.39 (1.07-1.79)**
		Digestive system cancer	73.7	0.02	R	0.03		**1.77 (1.05-2.96)**
		Other cancer	0	0.90	F	0.16		1.15 (0.94-1.41)
−1516G/T	GT *vs*. GG	Overall	32.7	0.19	F	<0.01	0.58	**1.99 (1.50-2.64)**
		Digestive system cancer	28.7	0.25	F	0.02		**1.77 (1.08-2.91)**
		Other cancer	54.1	0.11	R	0.01		**2.11 (1.26-3.56)**
	T *vs*. G	Overall	30.4	0.21	F	<0.01	0.58	**1.95 (1.48-2.58)**
		Digestive system cancer	25.7	0.26	F	0.02		**1.75 (1.08-2.85)**
		Other cancer	52.7	0.12	R	0.01		**2.07 (1.25-3.44)**
+4259T/G	TG *vs*. TT	Overall	59.1	0.03	R	<0.01	0.66	**2.21 (1.44-3.38)**
		Digestive system cancer	68.1	0.04	R	0.39		1.45 (0.62-3.41)
		Other cancer	0	0.85	F	<0.01		**2.87 (2.04-4.02)**
	G *vs*. T	Overall	59.8	0.03	R	<0.01	0.74	**2.14 (1.41-3.26)**
		Digestive system cancer	68.0	0.04	R	0.40		1.43 (0.62-3.29)
		Other cancer	0	0.85	F	<0.01		**2.77 (1.98-3.87)**

*P*_H:_ *P* value of heterogeneity test; *P*_Z:_ *P* value of Z test; *P*_E_: *P* value of Egger's test. R: random-effects model. F: fixed-effects model.

### Sensitivity analysis and publication bias

**Figure 3:**
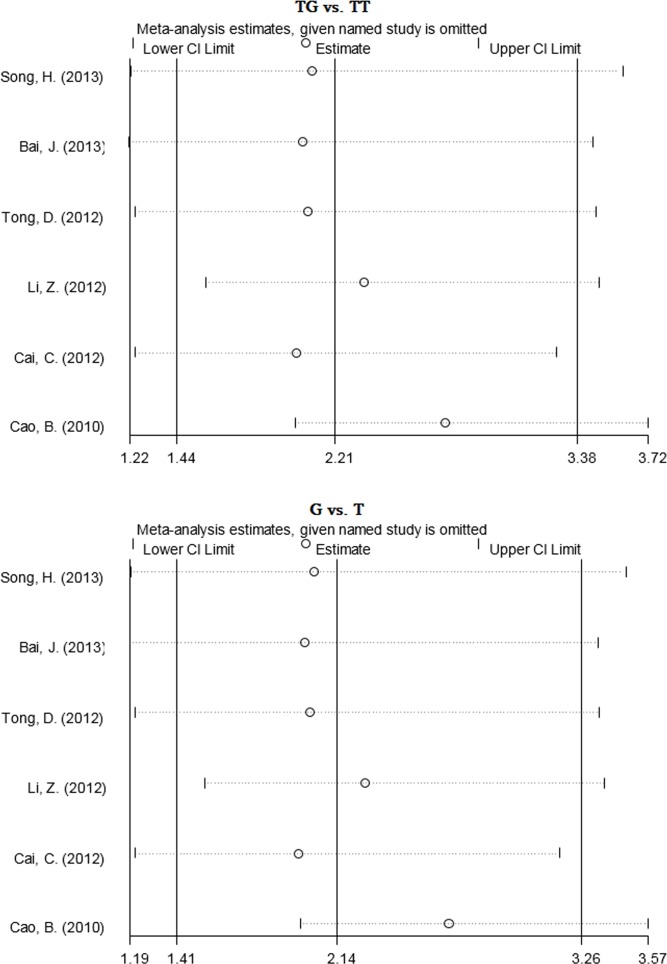
Sensitivity analysis of the pooled ORs and 95%CIs for *TIM-3* +4259T/G polymorphism

The sensitivity analysis showed that no single study altered the pooled ORs qualitatively, which provided the evidence of the stability of the meta-analysis (Figure 3, S3 and S4). Publication bias was assessed by Begg's test and Egger's test. As shown in Figure S5-S7, the shape of Begg's funnel plot did not reveal obvious asymmetry. However, Results of Egger's tests showed a borderline publication bias under the GT *vs*. GG model for *TIM-3* −1516G/T polymorphism (*P*E = 0.05), suggesting that the number of relevant studies may be insufficient (Table 2).

## DISCUSSION

A number of epidemiological studies have assessed the associations between *TIM-3* genetic polymorphisms (−1516G/T, −1516G/T, and +4259T/G) and the risk of different types of cancer. For instance, *Song H*, *et al*. found that the prevalence of *TIM-3* −574GT genotype and +4259TG genotype were significantly increased in the NHL cases than in controls [8]. *Bai J*, *et al*. confirmed that frequencies of TIM-3 +4259TG genotype were significantly different between the NSCLC cases and controls. Subjects carrying the +4259TG genotype had a 2.81-fold increased risk of NSCLC compared to those with the TT genotype [10]. There was also a report that showed a significant association between *TIM-3* −1516G/T polymorphism and the risk and distant metastasis of gastric cancer [7]. Compared to the carriers of *TIM-3* −1516GG genotype, the carriers of *TIM-3* −1516GT genotype had a 2.03-fold increased risk of gastric cancer [7]. These data suggest that *TIM-3* −1516G/T, −1516G/T, and +4259T/G polymorphisms are implicated in the development of cancer. However, there were also inconsistent results reported in the previous studies. For example, the *TIM-3* −1516G/T polymorphism did not reveal significant difference between NHL patients and healthy controls [8]. The *TIM-3* −1516G/T and −1516G/T polymorphisms did not show any correlation with NSCLC risk [10]. No association was observed between *TIM-3* +4259T/G polymorphism and gastric cancer [7]. In order to resolve this conflict, we conducted a meta-analysis on the association between three *TIM-3* polymorphisms (−1516G/T, −1516G/T, and +4259T/G) and cancer risk. Our results showed that *TIM-3* polymorphisms (−1516G/T, −1516G/T, and +4259T/G) were significantly associated with an increased risk of overall cancer in Chinese Han population. Subgroup analyses based on cancer system showed that *TIM-3* −1516G/T polymorphism was only associated with an increased risk of digestive system cancer in Chinese Han population. *TIM-3* −1516G/T polymorphism was associated with an increased risk of digestive system cancer and other cancer in Chinese Han population. *TIM-3* +4259T/G polymorphism was only associated with an increased risk of other cancer in Chinese Han population.

A large number of studies have confirmed that SNPs in cancer-related genes can contribute to individual susceptibility to cancer by affecting gene expression and function [14–15]. For instance, the −249T/C polymorphism in the promoter region of DEC1 gene reduced risk of squamous cell carcinoma of the head and neck by enhancing transcriptional activity of the DEC1 promoter and the DNA-protein-binding activity [14]. ERBB2 +2246A/G polymorphism (amino acid substitution: isoleucine to valine) is associated with an increased familial breast cancer risk. In addition, computational analyses showed that a substitution of isoleucine by a valine residue would stabilize the formation of active HER-2/NEU dimers [15]. Therefore, considering that TIM-3 can reduce the antigen-specific T cell responses and down-regulate the anti-tumor immunity in vivo by inhibiting the Th1 responses [16], we speculated that TIM-3 polymorphisms (−1516G/T, −1516G/T, and +4259T/G) conferred individual risk for cancer by increasing TIM-3 expression or enhancing TIM-3 activity.

Meta-analysis is a very powerful tool for analyzing cumulative data of studies where the individual sample sizes are small and the statistical power is low. To the best of our knowledge, no previous meta-analysis has comprehensively assessed the associations between the three SNPs and cancer risk. However, there are some limitations in the current meta-analysis. First of all, the number of published studies was not sufficiently large for a comprehensive analysis. Therefore, our analysis should be interpreted with caution, and more eligible studies on different types of cancer are needed. In addition, our results were based on unadjusted estimates because of lack of raw data including age, lifestyle, and environmental factors, which may cause a confounding bias.

In conclusion, our meta-analysis suggests that *TIM-3* polymorphisms (−1516G/T, −1516G/T, and +4259T/G) may increase an individual's susceptibility to cancer in Chinese Han population. However, more large-scale studies are warranted to confirm our finding in different cancer types.

## MATERIALS AND METHODS

### Search strategy

To identify eligible studies, we systematically searched PubMed, EMBASE and CNKI databases. The keywords used for search were as follows: “T-cell immunoglobulin- and mucin-domain-containing molecule 3 OR TIM-3”, “polymorphism OR variant” and “cancer OR carcinoma OR neoplasm”. There were no limitations on language and publication year. The last search was updated on December 12, 2015. Furthermore, references of all relevant articles were retrieved to identify additional eligible studies.

### Inclusion and exclusion criteria

Eligible studies must meet the following inclusion criteria: (a) case-control studies; (b) evaluating the association between *TIM-3* polymorphisms (−1516G/T, −1516G/T, and +4259T/G) and cancer risk; (c) available genotype frequencies; (d) the genotype distribution in control groups was in the Hardy-Weinberg equilibrium (HWE). Exclusion criteria were as follows: (a) letters, reviews, and case reports; (b) lack of genotype frequency data; (c) duplicate publication. In addition, if multiple studies had overlapping data, only those with complete data were included.

### Data extraction

Two authors independently selected the relevant articles and extracted the following data: first author's name, publication year, country, cancer type, genotyping methods, source of controls, number of cases and controls, genotype and allele frequency, and evidence of HWE in controls. Any disagreement was resolved by discussion between the authors.

### Statistical analysis

HWE in the control group of each study was examined by goodness-of-fit chi-square test, and *P*_HWE_ < 0.05 was considered as a deviation from HWE. The association between *TIM-3* polymorphisms (−1516G/T, −1516G/T, and +4259T/G) and cancer risk was evaluated by pooled OR and 95% CI. The significance of the pooled OR was assessed by the Z test, and *P*_Z_ < 0.05 was considered as statistically significant. The chi-square-based Q-test and I^2^ tests were used to investigate the heterogeneity between studies. If the *P*_H_ < 0.05 or I^2^ >50%, indicating the existence of between-study heterogeneity, the random-effects model was used to calculate the pooled ORs; otherwise, the fixed-effects model was applied to the analysis. Sensitivity analysis was carried out by sequentially omitting one study at a time to estimate the stability of the result. Publication bias among studies was determined using Begg's test and Egger's test, and *P*_E_ < 0.05 was considered significant. All statistical tests were performed with the STATA software (version 12.0; StataCorp, College Station, TX, USA).

## SUPPLEMENTARY MATERIAL FIGURES AND TABLES


