# Supplementary material for: Quantitative assessment of TIM-3 polymorphisms and cancer risk in Chinese Han population
Source: Oncotarget. 2016 Mar 17;7(24):35768–75. doi: 10.18632/oncotarget.8157 (PMC5094960; doi:10.18632/oncotarget.8157)
Supplement: Supplementary file 1 [file oncotarget-07-35768-s001.pdf]

Quantitative assessment of TIM-3 polymorphisms and cancer risk in Chinese Han population

Supplementary Material

Table S1: Genotype and allele frequencies of *TIM-3* polymorphisms in cases and controls.

Polymorphisms	First author	Year	Cancer system	Cases					Controls					P_{HWE}
				GG	GT	TT	G	T	GG	GT	TT	G	T	
TIM-3 -1516G/T	Song, H.	2013	Other cancer	411	85	0	907	85	432	80	0	944	80	0.06
	Li, Z.	2013	Digestive system cancer	216	52	3	484	58	290	27	1	607	29	0.66
	Bai, J.	2013	Other cancer	359	73	0	791	73	401	65	0	867	65	0.11
	Tong, D.	2012	Digestive system cancer	256	50	0	562	50	360	62	0	782	62	0.1
	Cai, C.	2012	Other cancer	268	54	0	590	54	342	60	0	744	60	0.11
	Cao, B.	2010	Digestive system cancer	176	34	2	386	38	229	23	0	481	23	0.45
				GG	GT	TT	G	T	GG	GT	TT	G	T	
TIM-3 -574G/T	Song, H.	2013	Other cancer	456	40	0	952	40	496	16	0	1008	16	0.72
	Bai, J.	2013	Other cancer	405	27	0	837	27	443	23	0	909	23	0.58
	Tong, D.	2012	Digestive system cancer	290	16	0	596	16	407	15	0	829	15	0.71
	Li, Z.	2012	Digestive system cancer	141	3	0	285	3	177	5	0	359	5	0.85
	Cai, C.	2012	Other cancer	294	28	0	616	28	389	13	0	791	13	0.74
	Cao, B.	2010	Digestive system cancer	192	20	0	404	20	243	9	0	495	9	0.77
				TT	TG	GG	T	G	TT	TG	GG	T	G	
TIM-3 +4259T/G	Song, H.	2013	Other cancer	451	45	0	947	45	493	19	0	1005	19	0.67
	Bai, J.	2013	Other cancer	385	47	0	817	47	447	19	0	913	19	0.65
	Tong, D.	2012	Digestive system cancer	279	27	0	585	27	408	14	0	830	14	0.73
	Li, Z.	2012	Digestive system cancer	143	1	0	287	1	178	4	0	360	4	0.88
	Cai, C.	2012	Other cancer	292	30	0	614	30	390	12	0	792	12	0.76
	Cao, B.	2010	Digestive system cancer	174	38	0	386	38	213	39	0	465	39	0.18

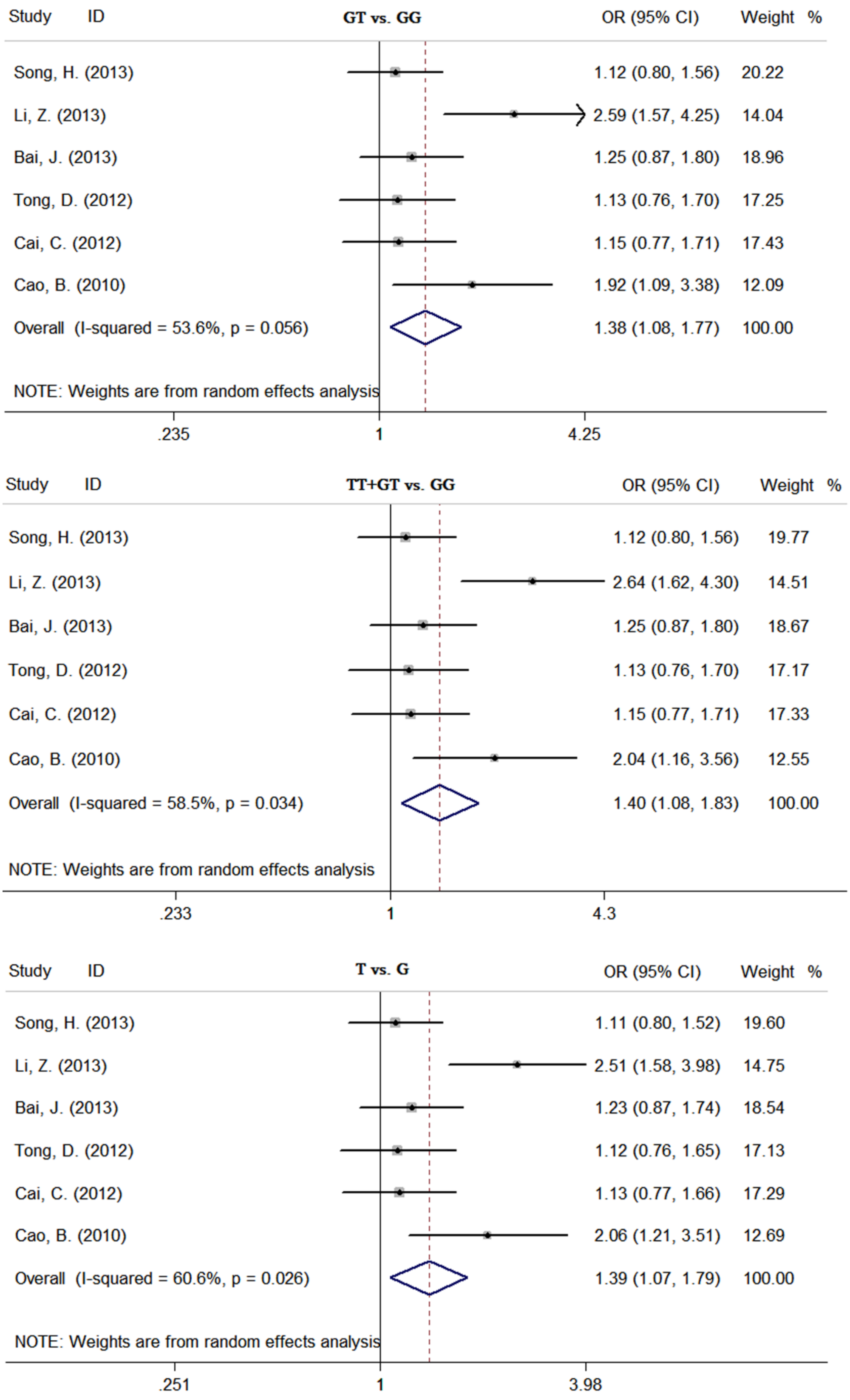

Figure S1: Forest plot of effect estimates for *TIM-3* -1516G/T polymorphism and overall cancer risk.

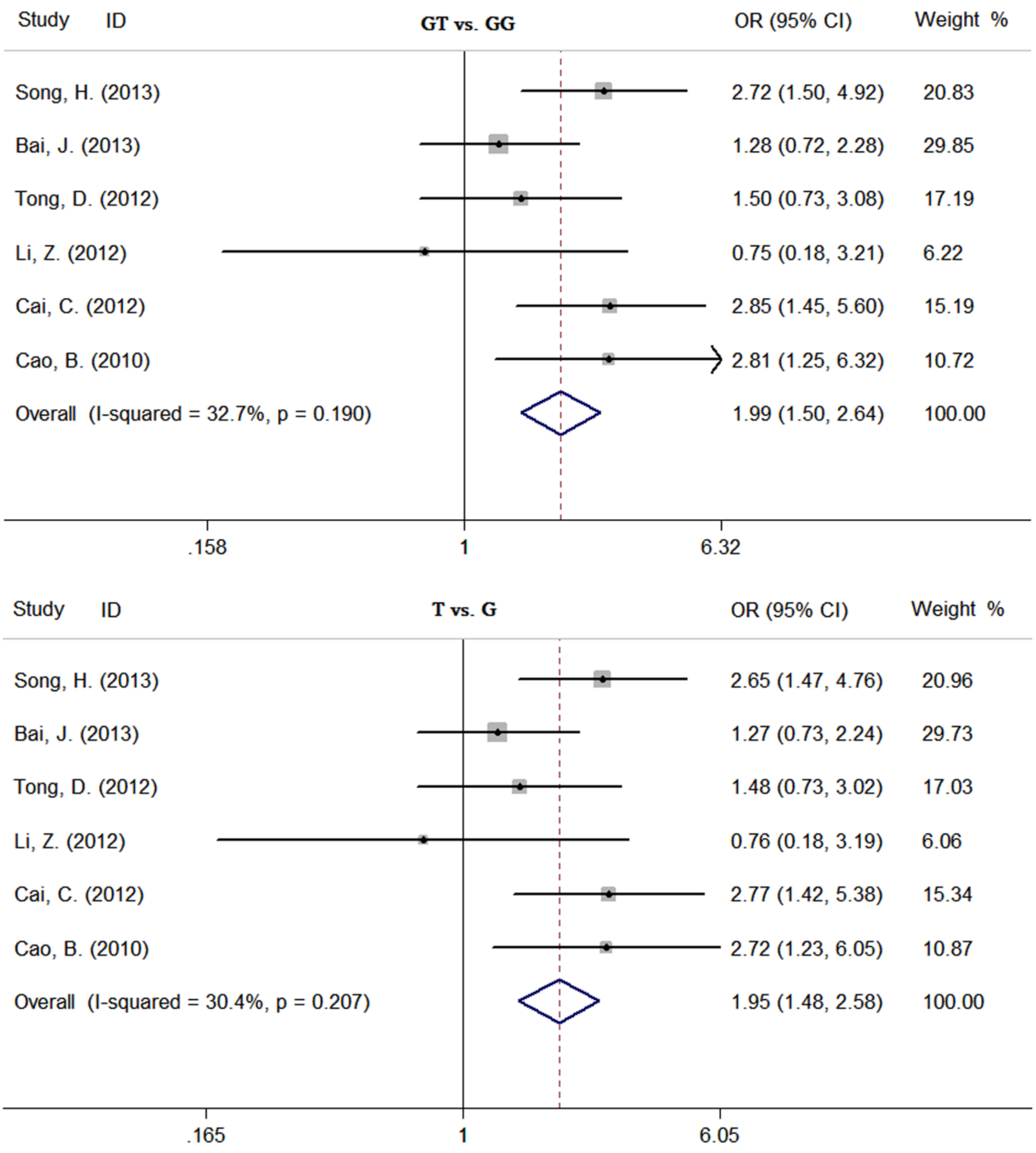

Figure S2: Forest plot of effect estimates for *TIM-3* -574G/T polymorphism and overall cancer risk.

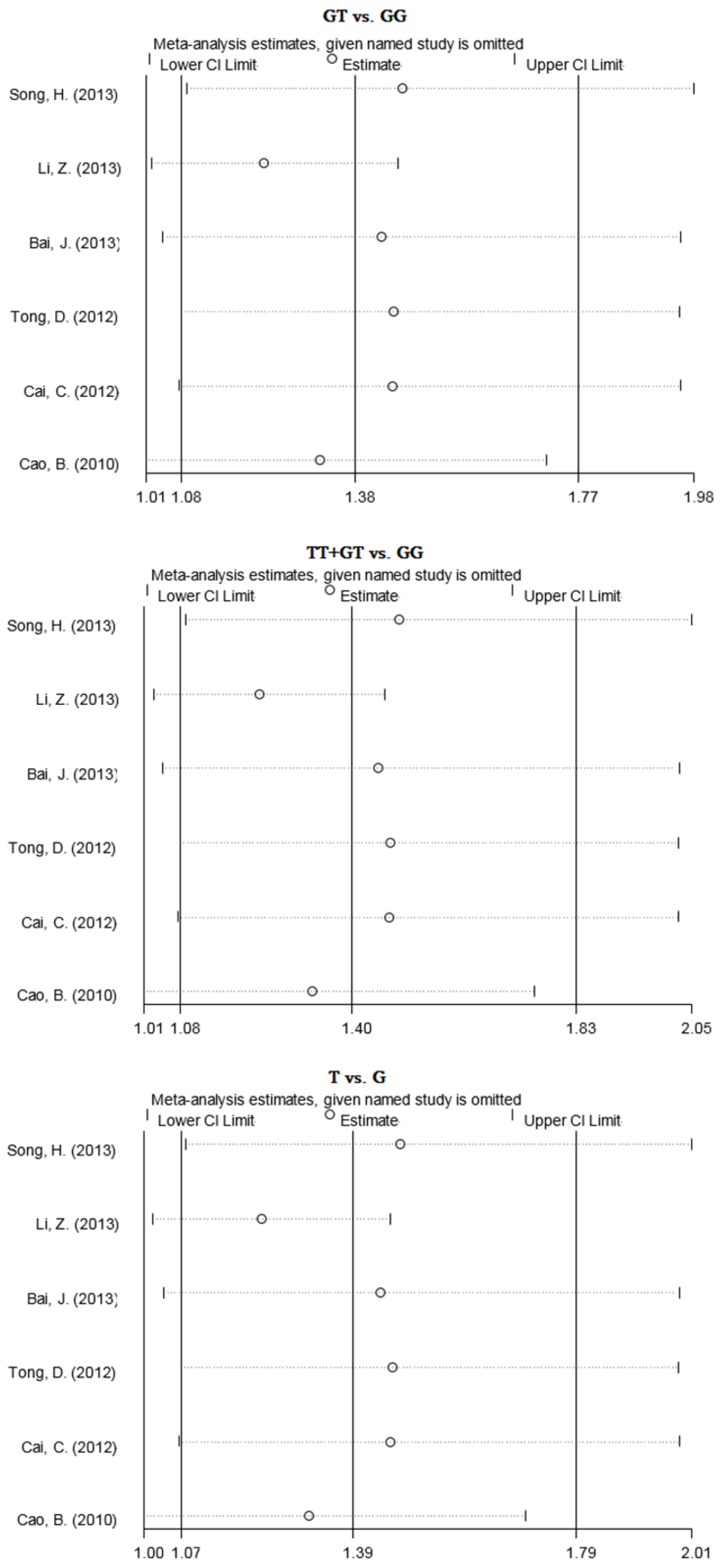

Figure S3: Sensitivity analysis of the pooled ORs and 95%CIs for *TIM-3* -1516G/T polymorphism.

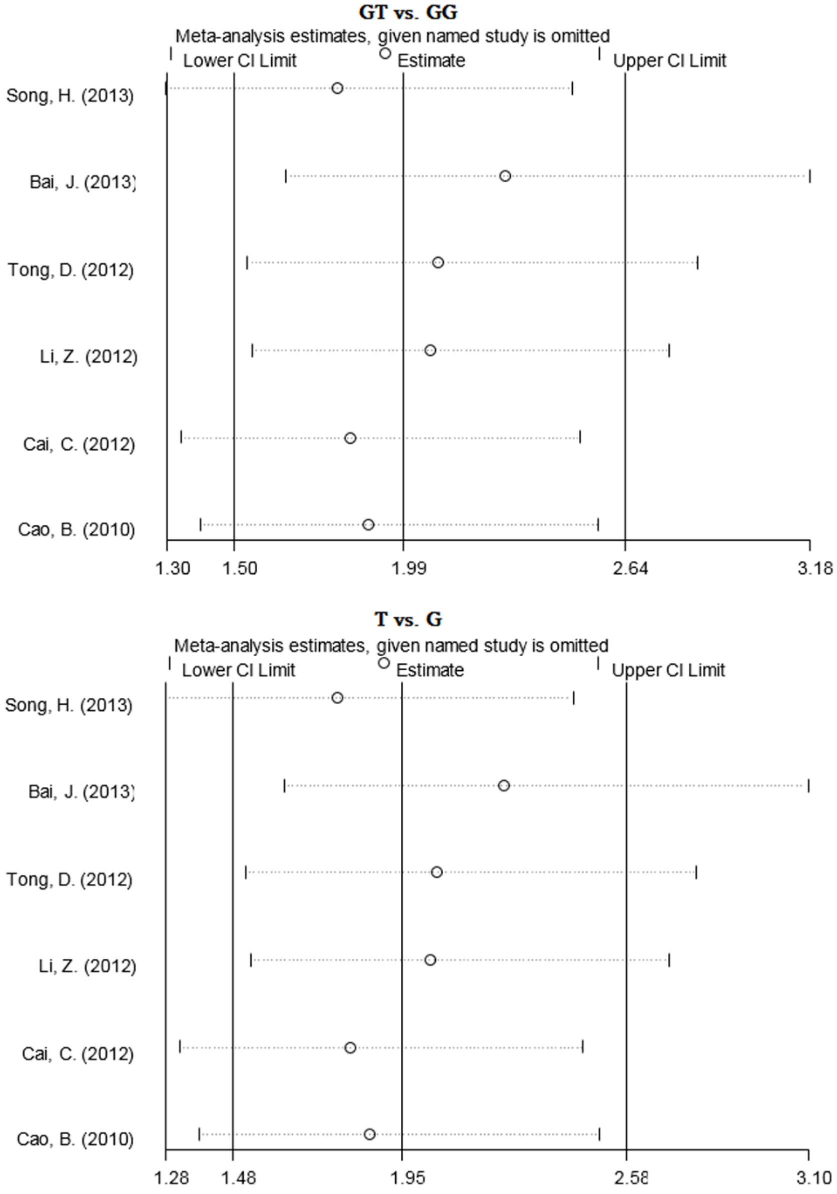

Figure S4: Sensitivity analysis of the pooled ORs and 95%CIs for *TIM-3* -574G/T polymorphism.

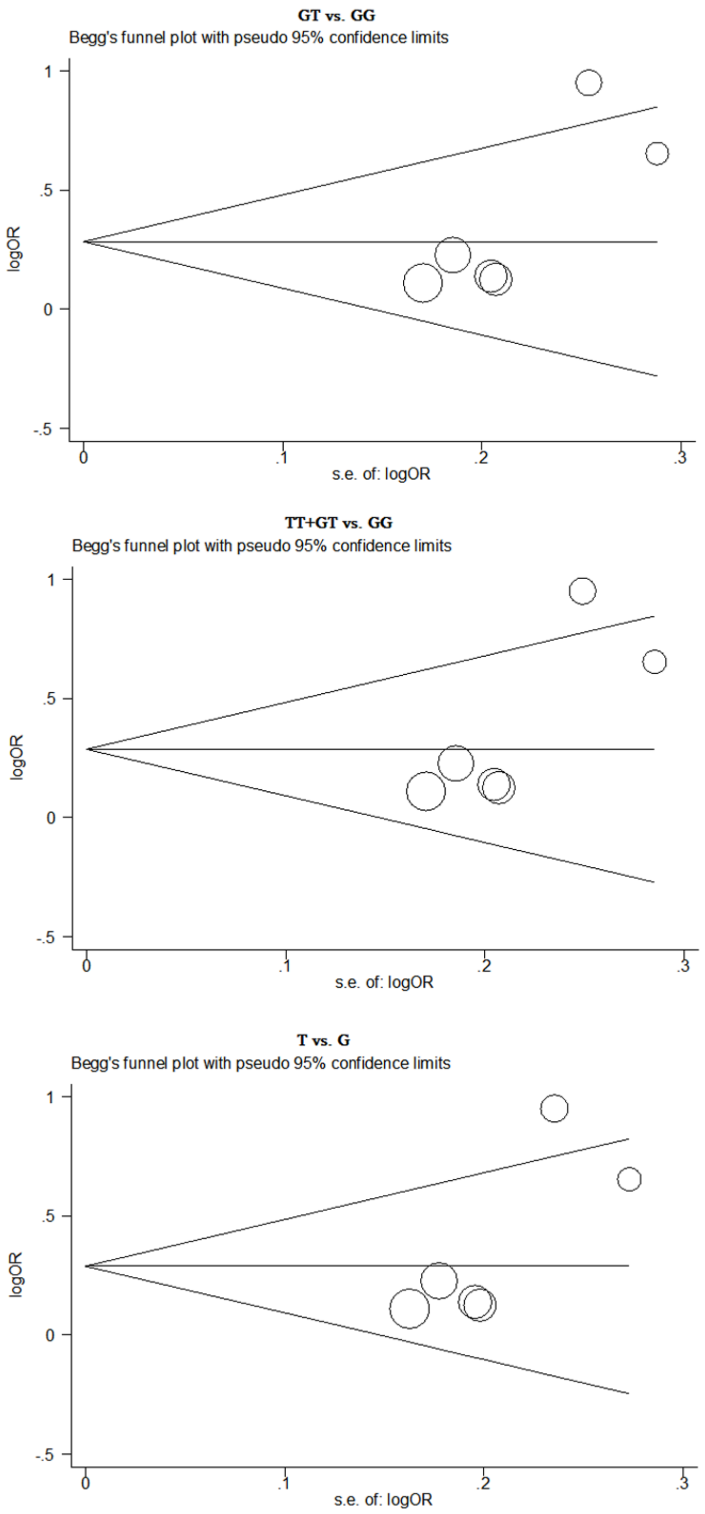

Figure S5: Funnel plot analysis of the association of *TIM-3* -1516G/T polymorphism with overall cancer risk.

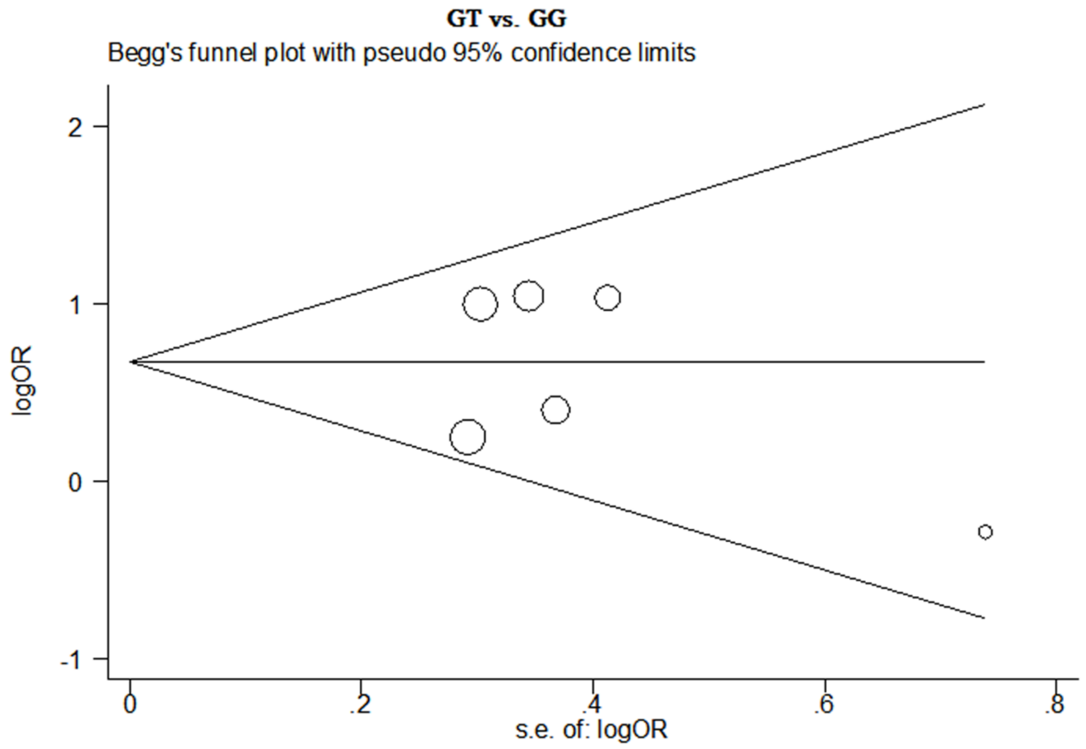

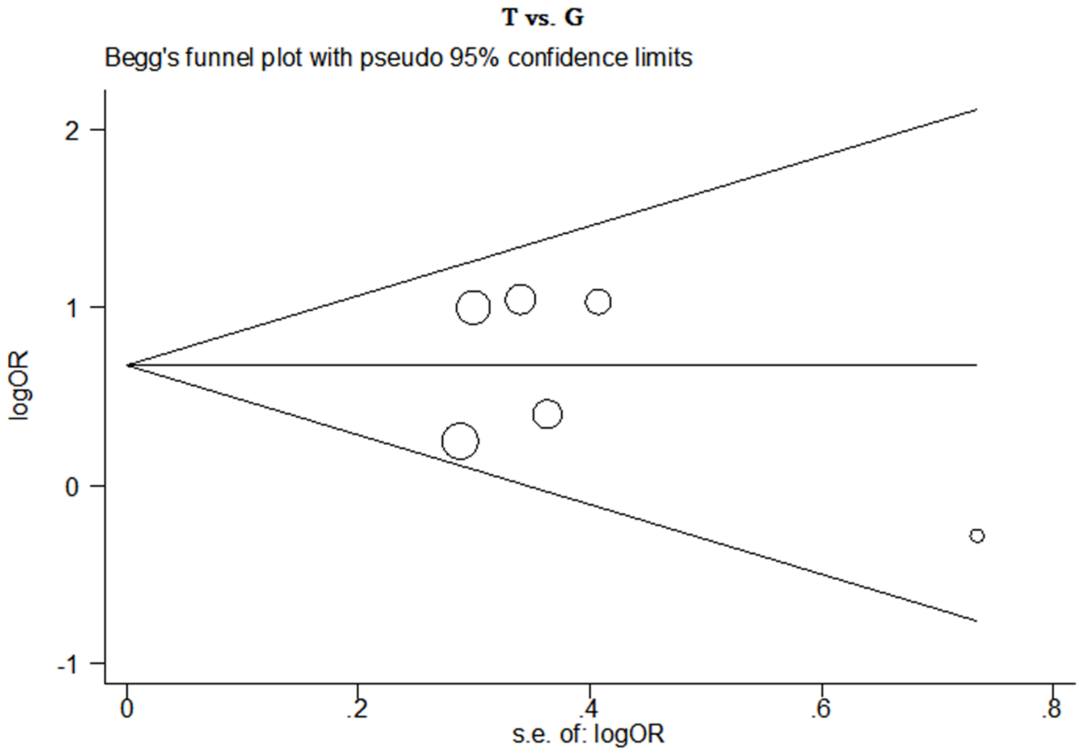

Figure S6: Funnel plot analysis of the association of *TIM-3* -574G/T polymorphism with overall cancer risk.

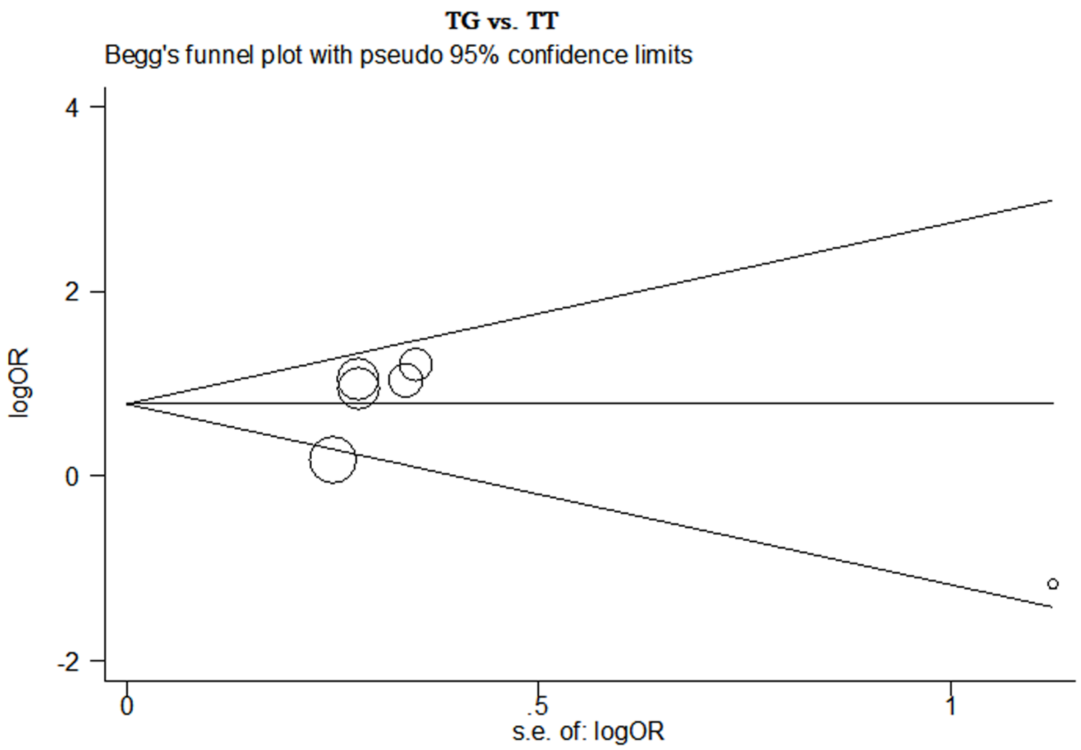

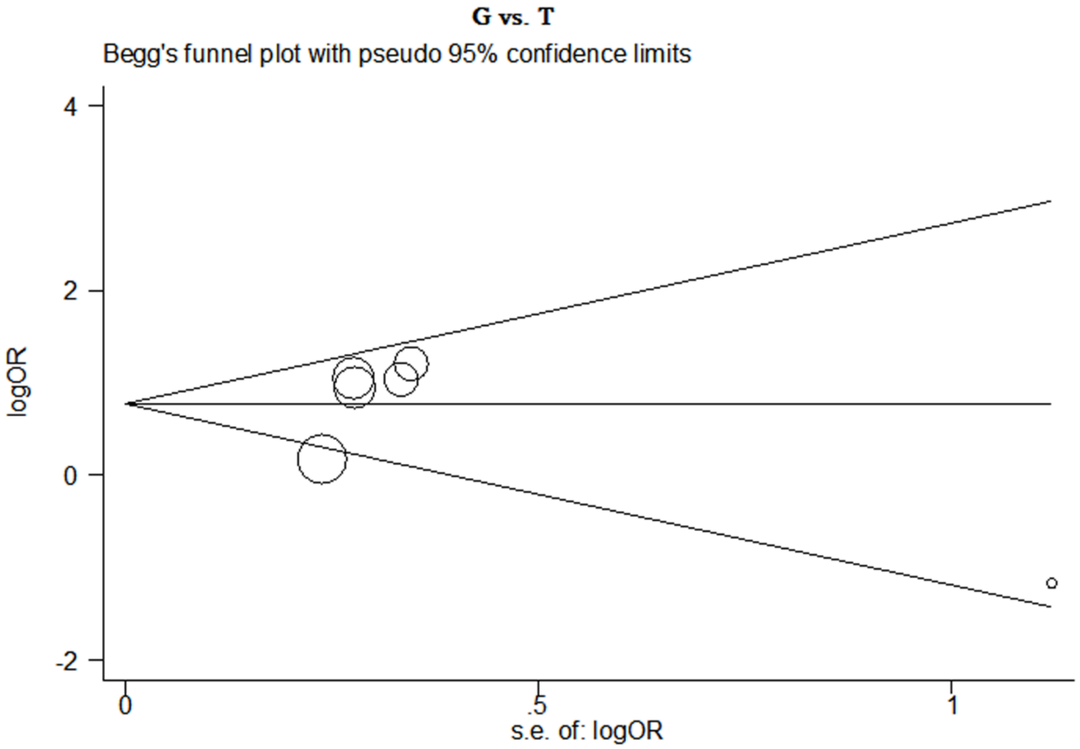

Figure S7: Funnel plot analysis of the association of *TIM-3* +4259T/G polymorphism with overall cancer risk.
